# Rectal Cancer Invasiveness: Whole-Lesion Diffusion-Weighted Imaging (DWI) Histogram Analysis by Comparison of Reduced Field-of-View and Conventional DWI Techniques

**DOI:** 10.1038/s41598-019-55059-0

**Published:** 2019-12-10

**Authors:** Yang Peng, Hao Tang, Xuemei Hu, Yaqi Shen, Ihab Kamel, Zhen Li, Daoyu Hu

**Affiliations:** 1 Department of Radiology, Tongji Hospital, Tongji Medical College, Huazhong University of Science and Technology, 1095 Jiefang Avenue, Wuhan, Hubei 430030 P.R. China; 2 Russell H. Morgan Department of Radiology and Radiological Science, The Johns Hopkins Medical Institutions, Baltimore, Maryland 21287 USA

**Keywords:** Rectal cancer, Gastrointestinal cancer

## Abstract

To explore the role of whole-lesion histogram analysis of apparent diffusion coefficient (ADC) for discriminating between T stages of rectal carcinoma by comparison of reduced field-of-view (FOV) and conventional DWI techniques. 102 patients with rectal cancer were enrolled in this retrospective study. All patients received preoperative MR scan at 3 T, including reduced and full FOV DWI sequences. Histogram parameters from two DWI methods were calculated and correlated with histological T stage of rectal cancer. The diagnostic performance of individual parameter for differentiating stage pT1-2 and pT3-4 tumors from both DWI techniques was assessed by receiver operating characteristic curve analysis. There were significant differences for the parameters of ADCmean, 50th, 75th, 90th, 95th percentiles, skewness and kurtosis of both DWI sequences in patients with pT1-2 as compared to those with pT3-4 tumors (P < 0.05), in addition to parameters including ADCmin (P = 0.015) and 25th percentile (P = 0.006) from rFOV DWI. Correlations were noted between T staging and above histogram parameters from rFOV DWI (r: −0.741–0.682) and fFOV DWI (r: −0.449–0.449), besides parameters of ADCmin (0.370) and 25th percentile (−0.425) from rFOV DWI. The AUCs of 75th and 90th percentiles from rFOV DWI were significantly higher than that from fFOV DWI (P = 0.0410 and P = 0.0208). The whole-lesion histogram analysis based on rFOV DWI was overall more advantageous than the one based on fFOV DWI in differentiating T staging of rectal cancer and the 90th percentile ADC from rFOV DWI was the value with the highest AUC (0.932).

## Introduction

Colorectal cancer is one of the most common cancers ranking the third in men and the second in women all over the world^1^ and is reported to have a locally recurrent rate of 3% to 20.6% after surgical resection^2,3^. The prognosis of rectal cancer is highly dependent on the early detection and accurate staging^4^. The utility of total mesorectal excision (TME) surgery and followed neoajuvant chemoradiotherapy has been the priority treatment for locally advanced rectal carcinoma (those with tumor invasion into perimesorectal fact and/or involvement of pelvic or mesorectal lymph nodes (cT3-4N0M0 or any T0N1M0)) with remarkable reduction of local recurrence rates^5,6^. However, excessive or insufficient treatment would lead to possible genitourinary function impairment and local recurrence^7,8^. Accurate T staging is essential to identify proper patients who are most likely to benefit from above combination treatment^9^.

MR imaging has been widely accepted as the optimum imaging modality for staging of rectal cancer and guiding treatment^10–13^. However, given to the utility of lower resolution MR techniques and thicker sections, discrimination of tumor invasion within muscular layer of rectum from tumor invasion by penetration through muscular layer even the whole rectal wall is sometimes challenging. More importantly, it is difficult to distinguish desmoplastic and inflammatory reactions from real tumor status because they all have similar invading behaviors of penetration through muscular rectal walls^14^. Therefore, improved MR techniques are needed for clinical management.

DWI is a noninvasive functional imaging tool for characterization of tumors, prognosis and response evaluation of different malignant cancers^11,15,16^. The ADC obtained from regions of interest (ROI) in DWI could provide quantitative information for cancerous tissues. As for rectal cancers, it has been reported that the ADC values were statistically significant between different T stages of tumors and ADC values could become an imaging biomarker reflecting tumor aggressiveness^17–19^. Moreover, the conventional DWI (fFOV DWI) technique, based on single-shot echo-planer imaging, can easily bring about distortions and artifacts^20^ because of its vulnerability to off-resonance due to narrow band width in the phase encoding direction. The reduced field-of-view (rFOV) DWI could provide DWI images with higher image quality and less artifacts, by employment of two-dimensional (2D) spatially-selective echo-planar radiofrequency (RF) pulse, followed by a 180° refocus pulse^21–24^. This technique could render the lesion margins and internal features of rectal cancer more conspicuously^25^.

While many studies reported mean ADC values from single slice-based ROIs, whole-lesion histogram analysis of ADC maps has been increasingly recognized for quantitative evaluation of intratumoral heterogeneity and tissue characteristics. One intrinsic feature of tumors is characterized by heterogeneity, which makes tumor tissue distinguished from normal tissue^26^. The whole-lesion method may better capture heterogeneous tumor areas with various diffusion characteristics demonstrated in the histogram and potential selective sampling bias could be decreased by measurement of whole tumor volumes^27^. The assessment of tumor heterogeneity has been reported to be highly promising for cancer diagnosis and treatment monitoring^28–30^. For instance, the ADC histogram analysis has been used for assessing aggressiveness of prostatic cancer^31^, or bladder cancer^32^ and discriminating adrenal adenoma from pheochromocytoma^33^. To date, the utility of whole-lesion histogram analysis based on rFOV DWI for preoperative T staging of rectal cancer has not been reported.

The purpose of this study was to explore the role of whole-lesion histogram analysis in assessment of histological T staging of rectal cancer by comparison of fFOV DWI and rFOV DWI techniques.

## Results

### Baseline characteristics

The baseline characteristics for patients and lesions of rectal cancers are shown in Table 1. All the 49 patients underwent surgery, and the histopathological results revealed 2 (4.1%) T1 stage, 19 (38.8%) T2 stage, 20 (40.8%) T3 stage and 8 (16.3%) T4 stage. There was significant difference between male age and female age (52.76 ± 11.27 vs. 64.00 ± 10.70, P = 0.002). Significant differences between male and female groups occurred in many histogram parameters based on rFOV DWI and conventional DWI, and the values of histogram parameters of female group were significantly higher than those of male group (P < 0.05) for both DWI techniques. But there was no significant difference between rFOV DWI and conventional DWI for the diagnostic efficacy of the same histogram parameter in relation to gender (P > 0.05). The tumor volume ranged from 0.94 cm^3^ to 31.38 cm^3^, and the mean tumor volume was 10.28 ± 6.84 cm^3^.

**Table 1:** Patient and tumor characteristics.

Variable	NO. Patient (%)
Age (years)	56.2 ± 12.2 (22–82)^a^
**Gender**	
Male	34 (69.4)
Female	15 (30.6)
**Distance of primary mass from anal verge**	
0–5.0 cm	23 (46.9)
5.1–10.0 cm	18 (36.7)
10.1–15.0 cm	8 (16.3)
**TNM stage**	
T category	
T1	2 (4.1)
T2	19 (38.8)
T3	20 (40.8)
T4	8 (16.3)
N category	
N0	31 (63.3)
N1/2	18 (36.7)
M category	
M0	43 (87.8)
M1	6 (12.2)
**Histological differentiation**	
Well	10 (20.4)
Moderately	15 (30.6)
Poorly	24 (49.0)
Tumor volume	10.28 ± 6.84 (0.94–31.38)^a^

^a^Mean ± SD (range);

Tumor volume is given in units of cm^3^.

### Interobserver and intraobserver variability

The histogram parameters of rFOV DWI technique had excellent interobserver and intraobserver agreement (P < 0.001 for each parameter). The histogram parameters of fFOV DWI technique showed good to excellent interobserver and intraobserver agreement (P < 0.001 for each parameter). As for the interobserver agreement, the ICC values ranged from 0.954 to 0.993 for rFOV DWI, and the ICC values ranged from 0.700 to 0.992 for fFOV DWI. As for intraobserver agreement, the ICC values ranged from 0.967 to 0.996 for rFOV DWI, and the ICC values ranged from 0.783 to 0.990 for fFOV DWI.

### Comparison of histogram parameters between T1-2 and T3-4 stages of rectal cancer based on rFOV DWI and fFOV DWI techniques

As for rFOV DWI, there was significant difference found in ADCmean, 75^th^ percentile ADC, 90^th^ percentile ADC, 95^th^ percentile ADC, skewness, kurtosis (P < 0.001 for all), ADCmin (P = 0.015), 25^th^ percentile ADC (P = 0.006) and 50^th^ percentile ADC (P = 0.001). As for fFOV DWI, significant difference was found in 75^th^ percentile ADC, 90^th^ percentile ADC, skewness, kurtosis (P = 0.001 for all), ADCmean, 95^th^ percentile ADC (P = 0.011 for both), and 50^th^ percentile ADC (P = 0.008) (Table 2). The difference of other parameters such as ADCmax, 5^th^, 10^th^ percentiles, tumor volume and entropy was not significant for both DWI techniques.

**Table 2:** Comparison of whole-lesion histogram parameters between T1-2 and T3-4 stages of rectal cancer based on rFOV DWI and fFOV DWI techniques.

Parameter /DWI technique	T1-2 (N = 21)	T3-4 (N = 28)	P-value	Correlation coefficient (T staging)	P-value
**ADCmean**					
rFOV DWI	1198.55 ± 138.55	1057.30 ± 59.76	<0.001	−0.601 (−0.783, −0.373)	<0.001
fFOV DWI	1163.26 ± 144.97	1076.50 ± 82.67	0.011	−0.309 (−0.549, −0.027)	0.031
**ADCmin**					
rFOV DWI	188.33 ± 169.46	313.57 ± 173.79	0.015	0.370 (0.106, 0.608)	0.009
fFOV DWI	423.57 ± 264.64	546.25 ± 277.76	0.125	0.155 (−0.127, 0.403)	0.289
**25**^**th**^ **percentile ADC**					
rFOV DWI	956.73 ± 122.48	882.37 ± 55.72	0.006	−0.425 (−0.662, −0.132)	0.002
fFOV DWI	963.04 ± 141.83	907.32 ± 74.54	0.081	−0.223 (−0.504, 0.093)	0.123
**50**^**th**^ **percentile ADC**					
rFOV DWI	1154.88 ± 150.04	1017.95 ± 58.61	0.001	−0.582 (−0.770, −0.334)	<0.001
fFOV DWI	1122.62 ± 153.13	1028.84 ± 83.06	0.008	−0.356 (−0.575, −0.105)	0.012
**75**^**th**^ **percentile ADC**					
rFOV DWI	1408.10 ± 158.13	1184.69 ± 72.15	<0.001	−0.726 (−0.825, −0.568)	<0.001
fFOV DWI	1329.35 ± 155.40	1196.34 ± 100.23	0.001	−0.449 (−0.654, −0.190)	0.001
**90**^**th**^ **percentile ADC**					
rFOV DWI	1653.76 ± 191.15	1388.86 ± 93.16	<0.001	−0.741 (−0.837, −0.573)	<0.001
fFOV DWI	1545.81 ± 176.32	1396.41 ± 126.94	0.001	−0.424 (−0.633, −0.152)	0.002
**95**^**th**^ **percentile ADC**					
rFOV DWI	1807.04 ± 227.21	1550.63 ± 122.67	<0.001	−0.647 (−0.787, −0.454)	<0.001
fFOV DWI	1689.75 ± 212.16	1543.65 ± 172.10	0.011	−0.362 (-0.604, −0.091)	0.011
**Skewness**					
rFOV DWI	0.65 ± 0.40	1.18 ± 0.43	<0.001	0.560 (0.321, 0.734)	<0.001
fFOV DWI	0.70 ± 0.60	1.25 ± 0.48	0.001	0.423 (0.151, 0.653)	0.002
**Kurtosis**					
rFOV DWI	0.88 ± 0.94	3.26 ± 2.04	<0.001	0.682 (0.514, 0.806)	<0.001
fFOV DWI	1.28 ± 1.21	3.01 ± 2.15	0.001	0.499 (0.243, 0.688)	<0.001

Note. — data are means and standard deviations (averages between readers).

ADC values are given in units of 10^−6^ mm^2^/s.

Data in parentheses are 95% confidence intervals.

**Figure 1:**
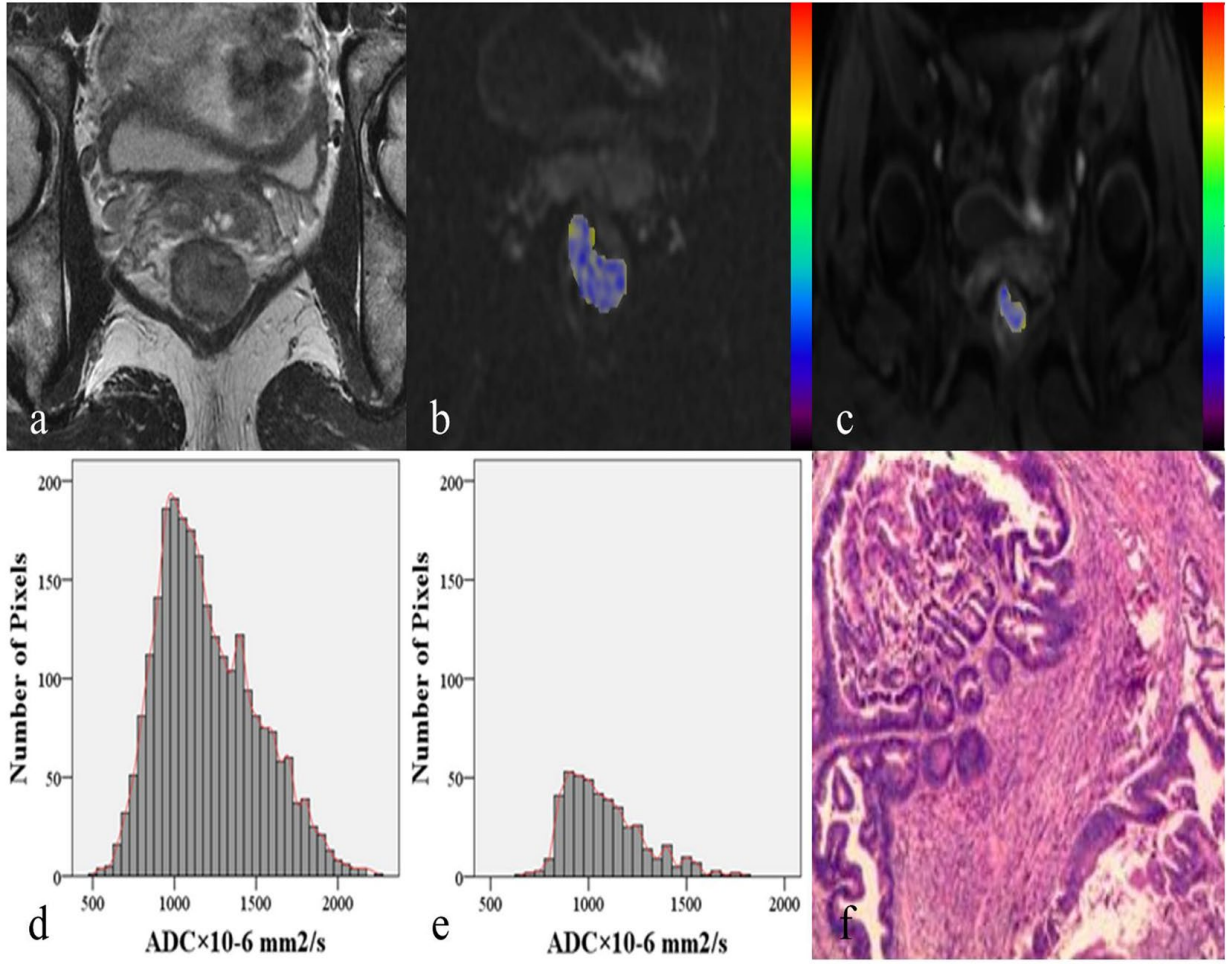
A 46-year-old female patient with T2 stage rectal adenocarcinoma. (**a**) Axial T2-weighted image showing intra-luminal lesion with intermediate signal intensity in the lower segment of rectum. (**b**,**c**) Corresponding diffusion-weighted images of rFOV DWI and fFOV DWI with the identical lesion for reconstruction of ADC measurements. (**d**,**e**) Whole-lesion histograms of rFOV DWI and fFOV DWI. F: Histopathological H&E (hematoxylin & eosin staining, ×100) image shows a moderately-differentiated adenocarcinoma invading the muscular layer of rectal wall.

**Figure 2:**
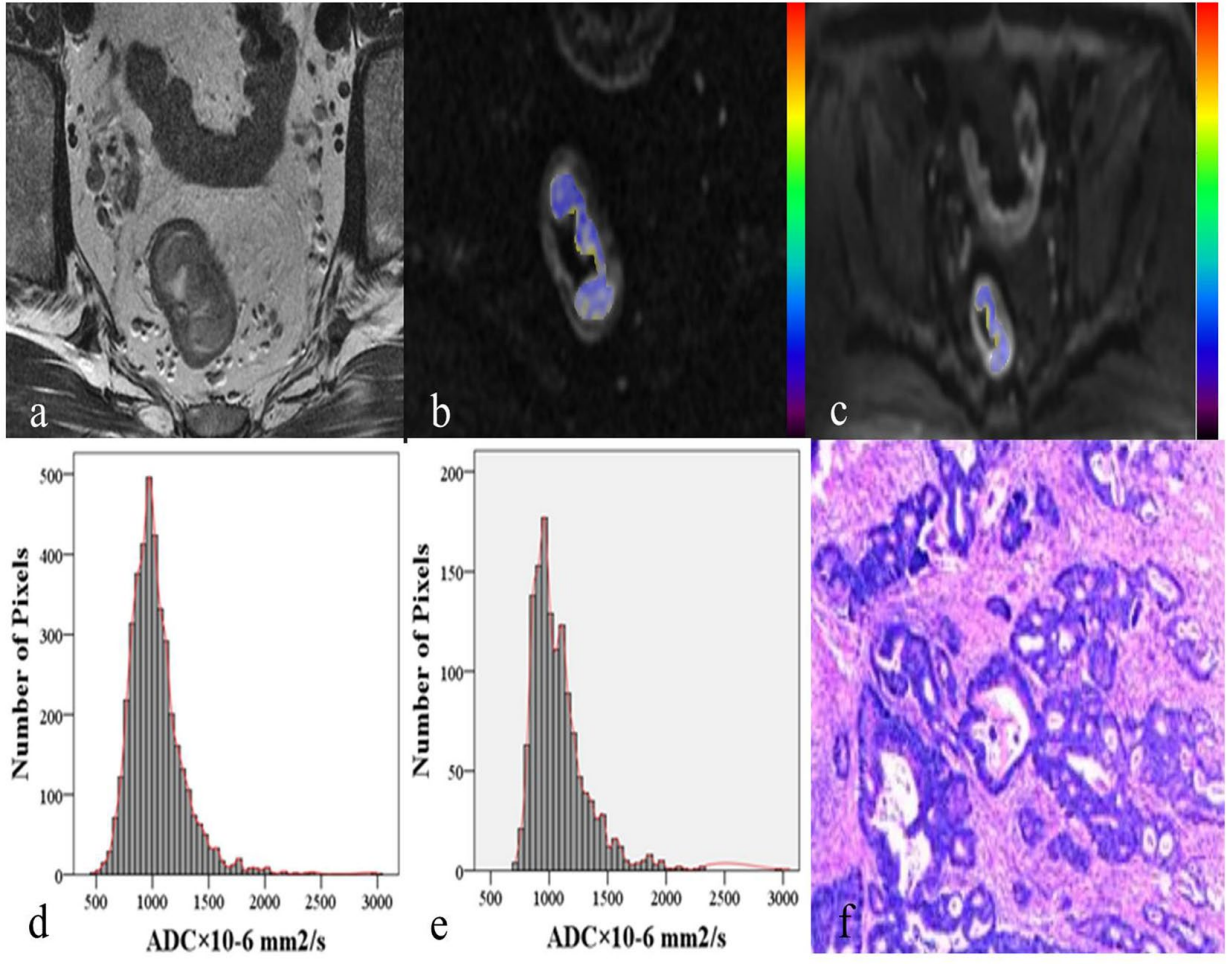
A 42-year-old male patient with T3 stage rectal adenocarcinoma. (**a**) Axial T2-weighted image showing irregular thickening of rectal wall with intra-luminal mass in the upper segment of rectum. (**b**,**c**) Corresponding diffusion-weighted images of rFOV DWI and fFOV DWI with the same lesion for reconstruction of ADC measurements. (**d**,**e**) Whole-lesion histograms of rFOV DWI and fFOV DWI. (**f**) Histopathological H&E (hematoxylin & eosin staining, ×100) image shows a moderately-differentiated adenocarcinoma invading the whole layer of rectal wall.

With respect to the parameters from rFOV DWI, there were significant correlations between T staging and following parameters (Table 2): ADCmean, 50^th^ percentile ADC, 75^th^ percentile ADC, 90^th^ percentile ADC, 95^th^ percentile ADC, skewness, kurtosis (P < 0.001), ADCmin (P = 0.009), and 25^th^ percentile ADC (P = 0.002). Among the parameters, 90^th^ percentile ADC demonstrated the strongest correlation of −0.741 with T staging (95% CI: −0.837, −0.573), while kurtosis showed the highest correlation of 0.682 with T staging (95% CI: 0.514, 0.806). There was also a positive correlation found between ADCmin and T staging (0.370; 95% CI: 0.106, 0.608). As for the parameters from fFOV DWI, significant correlations were observed between T staging and following parameters: ADCmean (P = 0.031), 50^th^ percentile ADC (P = 0.012), 75^th^ percentile ADC (P = 0.001), 90^th^ percentile ADC (P = 0.002), 95^th^ percentile ADC (P = 0.011), skewness (P = 0.002), and kurtosis (P < 0.001). 75^th^ percentile ADC had the relatively strongest correlation of −0.449 with T staging (95% CI: −0.654, −0.190), while kurtosis showed the highest correlation of 0.449 with T staging (95% CI: 0.243, 0.688). Representative cases from two DWI techniques to demonstrate different T staging of rectal cancer are shown in Figs. 1 and 2 respectively.

### Comparison of ROC analysis of parameters for T1-2 vs. T3-4 stage tumors based on rFOV DWI and fFOV DWI techniques

**Figure 3:**
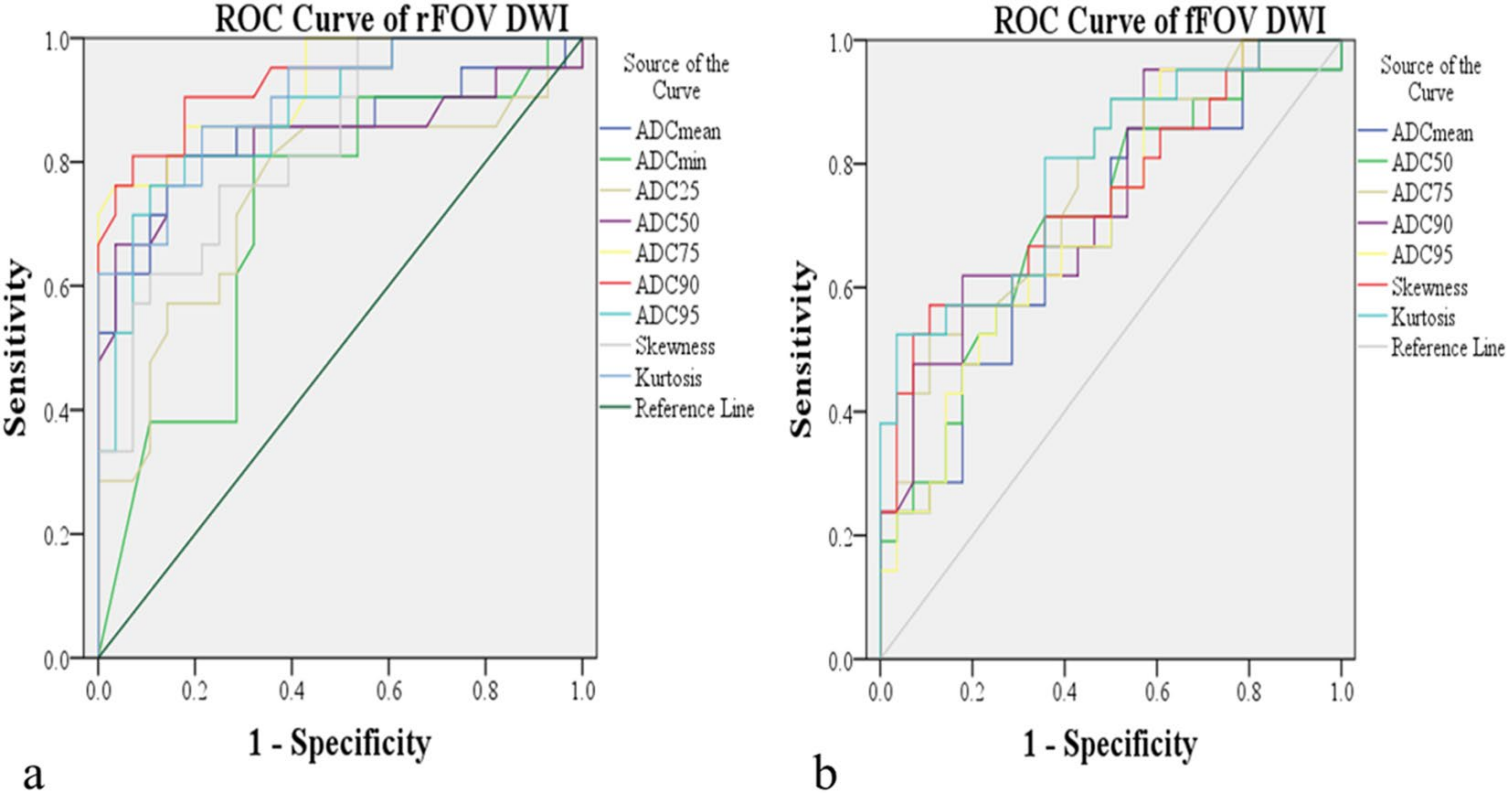
Receiver operating characteristic (ROC) curves demonstrating the false positive rate (sensitivity) and true positive rate (specificity) of whole-lesion histogram parameters for distinguishing pT1-2 and pT3-4 stages of rectal cancer based on rFOV DWI and fFOV DWI techniques. The areas under the ROC curve (AUC) of rFOV DWI (**a**) for ADCmean, ADCmin, the 25^th^, 50^th^, 75^th^, 90^th^, and 95^th^ percentiles of ADC values, skewness and kurtosis were 0.850, 0.714, 0.747, 0.839, 0.923, 0.932, 0.878, 0.827 and 0.898 respectively. The areas under the ROC curve (AUC) of fFOV DWI (**b**) for ADCmean, 50^th^, 75^th^, 90^th^, and 95^th^ percentiles of ADC values, skewness and kurtosis were 0.680, 0.707, 0.762, 0.747, 0.711, 0.747, and 0.791 respectively.

According to the ROC analysis test from rFOV DWI, the 90^th^ percentile ADC demonstrated the highest AUC of 0.932 (95% CI: 0.860, 1.000) in distinguishing pT1-2 and pT3-4 stages of rectal cancer, whereas ADCmin achieved the the lowest AUC of 0.714 (95% CI: 0.565, 0.863). The optimal cutoff value of the 90^th^ percentile ADC was 1527.50 × 10^−6^ mm^2^/s with sensitivity of 81.00% and specificity of 92.90%. As for fFOV DWI, kurtosis achieved the highest AUC of 0.791 (95% CI: 0.663, 0.919) in distinguishing pT1-2 and pT3-4 stages of rectal cancer. However, ADCmean had the lowest AUC of 0.680 (95% CI: 0.527, 0.834). The optimal cutoff value of kurtosis was 0.984 with sensitivity of 96.40% and specificity of 52.40% (Table 3, Fig. 3). The AUCs of 75^th^ percentile ADC (0.923 vs.0.762, P = 0.0410) and 90^th^ percentile ADC (0.932 vs. 0.747, P = 0.0208) from rFOV DWI were significantly higher than that from fFOV DWI.

**Table 3:** Comparison of ROC analysis of whole-lesion histogram parameters for T1-2 vs. T3-4 stage tumors based on rFOV DWI and fFOV DWI techniques.

Parameter/DWI technique	Cutoff	Sensitivity (%)	Specificity (%)	Area under the curve (AUC)	P-value
**ADCmean**					
rFOV DWI	1129.06	81.00	85.70	0.850 (0.728, 0.973)	<0.001
fFOV DWI	1057.87	85.70	46.40	0.680 (0.527, 0.834)	0.032
**ADCmin**					
rFOV DWI	287.50	67.90	81.00	0.714 (0.565, 0.863)	0.011
fFOV DWI	562.50	52.40	50.00	0.590 (0.427, 0.753)	0.284
**25**^**th**^ **percentile ADC**					
rFOV DWI	892.50	81.00	64.30	0.747 (0.598, 0.897)	0.003
fFOV DWI	945.00	52.40	71.40	0.630 (0.468, 0.792)	0.122
**50**^**th**^ **percentile ADC**					
rFOV DWI	1090.00	81.00	85.70	0.839 (0.708, 0.970)	<0.001
fFOV DWI	1057.50	71.40	64.30	0.707 (0.558, 0.857)	0.014
**75**^**th**^ **percentile ADC**					
rFOV DWI	1310.00	76.20	96.40	0.923 (0.849, 0.998)	<0.001
fFOV DWI	1310.00	52.40	89.30	0.762 (0.628, 0.896)	0.002
**90**^**th**^ **percentile ADC**					
rFOV DWI	1527.50	81.00	92.90	0.932 (0.860, 1.000)	<0.001
fFOV DWI	1502.50	61.90	82.10	0.747 (0.609, 0.886)	0.003
**95**^**th**^ **percentile ADC**					
rFOV DWI	1691.00	76.20	89.30	0.878 (0.781, 0.974)	<0.001
fFOV DWI	1460.88	95.20	39.30	0.711 (0.567, 0.855)	0.012
**Skewness**					
rFOV DWI	0.932	75.00	76.20	0.827 (0.713, 0.940)	<0.001
fFOV DWI	0.760	89.30	57.10	0.747 (0.604, 0.889)	0.003
**Kurtosis**					
rFOV DWI	1.740	78.60	85.70	0.898 (0.812, 0.984)	<0.001
fFOV DWI	0.984	96.40	52.40	0.791 (0.663, 0.919	0.001

Note. — Data in parentheses are 95% confidence intervals.

ADC values are given in units of 10^−6^ mm^2^/s.

## Discussion

In our study, we investigated the role of rFOV DWI and fFOV DWI techniques in assessment of pathological T staging of rectal cancer by using whole-lesion histogram analysis. The results demonstrated that the histogram parameters from both DWI techniques could be utilized to discriminate between pT1-2 and pT3-4 stages of rectal cancer including ADCmean, 50^th^, 75^th^, and 95^th^ percentile ADCs, skewness and kurtosis, besides the parameters of ADCmin and 25^th^ percentile ADC from rFOV DWI. The AUCs of 75^th^ and 90^th^ percentile ADCs from rFOV DWI were significantly higher than that from fFOV DWI (P = 0.0410 and P = 0.0208 respectively) . The 90^th^ percentile ADC from rFOV DWI achieved the highest AUC (0.932) in differentiating T staging of rectal cancer, with the optimal cutoff value of 1527.50 × 10^−6^ mm^2^/s. The whole-lesion histogram analysis based on rFOV DWI was more advantageous over that based on fFOV DWI in evaluation of rectal cancer invasiveness.

We also assessed the interobserver and intraobserver variability for whole-lesion histogram parameters from both two DWI techniques. The results showed overall good to excellent agreement. The excellent interobserver and intraobserver variability confirmed relatively better reproducibility and stability of whole-lesion histogram analysis in combination with rFOV DWI over fFOV DWI in assessment of rectal cancer.

The statistical significance was found in histogram parameters such as ADCmean, 50^th^, 75^th^, 90^th^, and 95^th^ percentile ADCs in assessment of histopathological T staging for both DWI techniques in our study. It was mainly presided by higher percentile ADC in relation to T staging. Some previous studies had similar results, which were consistent with our finding. For instance, both Takahashi *et al*. and Kang *et al*. reported that high percentile ADCs were significantly effective in the differential diagnosis of uterine neoplasm and grading of gliomas^34,35^. On the other side, Zhang *et al*. and Donati *et al*. reported that the low percentile (5^th^, 10^th^, and 25^th^) ADCs exhibited better diagnostic performances in grading of gastric cancer and prostate cancer aggressiveness^31,36^. Above investigations demonstrated histogram parameters were useful for histopathological characterization of tumor grading and invasiveness. The low or high percentile ADCs played different roles in assessment of tumor biological features from diverse organs. As for rectal cancer, high percentiles ADCs were found to be significantly meaningful in evaluation of T staging. The low percentile ADCs represented areas with higher cellularity and less water restriction, however, the high percentile ADCs indicated areas with necrotic/cystic components, and less water molecules^37,38^. One probable explanation for the findings in our study was that rectal cancers with pT3-4 stage often contain many invisible small areas of necrosis and tiny cystic changes, although the conspicuous necrotic and cystic regions were excluded for histogram analysis. The cystic and necrotic components may result in the high frequency of high ADC voxels in contrast to pT1-2 stage tumors, rendering the differentiation between two stages possible.

Moreover, 25^th^ percentile ADC and ADCmin from rFOV DWI were also significantly correlated with histological T staging of rectal cancer. The 25^th^ percentile ADC might indicate the superiority of rFOV DWI over fFOV DWI in detecting regions for tumors with focal areas of high cellularity, which were represented by low percentile ADC^31^. As for the parameter of ADCmin from rFOV DWI, it was correlated positively with T staging. Liu *et al*. found that there was no significant correlation between minimum ADC and T staging of rectal cancer by texture analysis^5^, which was consistent with our finding from fFOV DWI. The proved correlation of ADCmin from rFOV DWI with T stage might be explained by the technical advantage of rFOV DWI over fFOV DWI. The rFOV DWI technique provided images with less distortions and ghosts, rendering anatomic structures of lesions more conspicuous. However, in contrast to our finding from rFOV DWI, Kang *et al*. reported minimum ADC correlated inversely with grading of gliomas^35^. The different finding of ADCmin may be due to the internal features of tumor itself and DWI technique applied. Further investigation is needed to find the actual relationship between ADCmin and histological T staging of rectal cancer.

We considered pT1-2 stage rectal cancer was associated with lower skewness and kurtosis, in contrast to pT3-4 stage for both DWI techniques. Theoretically, skewness represents the asymmetry of distribution of ADC values, while kurtosis reflects the peakedness of ADC distribution^33^. Higher values of skewness and kurtosis are often associated with more complexities of different components in a given ROI and increased heterogeneity of lesion tissues^5^. The higher-stage rectal tumor was often characterized by more heterogeneous tissue condition and cellular components. This may explain why there were significantly higher kurtosis and skewness in pT3-4 stage than that in pT1-2 stage. The previous study reported comparatively lower value of correlation coefficients for kurtosis (−0.126) and skewness (0.277) in a texture analysis of rectal cancer^5^. The higher results in our study can be explained by the ROI selection method. In our study, the acquisition of histogram parameters was based on whole-lesion covering the whole tumor volume, while the previous texture study was based on single-slice method. The whole-volume method can be more reliable than single-slice method, because it excludes the possibility of variations in selection of single slice^11,39^.

As for the parameter of entropy, it did not differ significantly between stages of rectal cancer. Entropy refers to statistical measurement of irregularity of gray-level signal intensity (SI) within the volume of interest. Theoretically, higher value of entropy indicates more random distribution of SI and heterogeneity^40^. The negative finding in our study may be caused by relatively small number of patients for each stage of rectal cancer. More patients are needed to verify the significance of entropy in relation to T staging.

In addition, our research found the spearman correlation coefficients demonstrated that the histogram parameters (ADCmean, 50^th^, 75^th^, 90^th^, and 95^th^ percentile ADCs) from both DWI techniques correlated inversely with histological T staging. The histogram metrics from rFOV DWI presented with higher correlation coefficients, which demonstrated superiority of rFOV DWI over fFOV DWI by comparison of above parameters individually. The variability of correlation difference may be mainly caused by utility of different DWI techniques. The rFOV DWI has been reported to provide comparatively good quality and high-resolution images of small structures of rectal lesions over fFOV DWI. The rFOV DWI technique can enlarge the bandwidth along the phase-encoding direction and shorten the readout time, therefore improving the DWI image quality with fewer distortions and susceptibility artifacts.

### Limitations

Our study had limitations. First, this was a retrospective study with inevitable biases for patient selection. Second, the sample size was comparatively small, especially for the pT1 stage of rectal cancer. The final result might not reflect the real status of pT1-2 stage. Third, only b values of 0 and 800 s/mm^2^ were applied for analysis. The finding of optimal maximum b value and utility of intravoxel incoherent motion are needed to improve the results of ADC quantification. Fourth, the stratification of patients into T1-T2 or T3-T4 group was not entirely prognostic relevant and not relevant from recent treatment guidelines, which should be mentioned in our study. Finally, according to the latest ESMO (European Society for Medical Oncology) guidelines, patients with cT3a/bN0M0 tumors located in the middle or upper third of the rectum and cT3a/bN1M0 tumors located in the upper third of the rectum could be recommended for major TME surgery without preoperative RT (radiotherapy) or CRT (chemoradiotherapy) treatment. Moreover, as for some patients with cT4aN0M0 tumors located in the upper third of the rectum, ESMO guidelines do not explicitly indicate mandatory preoperative chemoradiation treatment. Besides, some patients with advanced rectal cancer underwent operations because they had complications such as intestinal obstruction or bleeding. Above were the reasons for the enrolled patients with T3 or higher stage of rectal cancer in our hospital having surgical operations without preoperational neoajuvant treatment.

## Conclusion

Our study suggests that whole-lesion histogram analyses of both rFOV DWI and fFOV DWI techniques could help assess rectal cancer invasiveness. The rFOV DWI technique performs better than fFOV DWI technique in assessment of rectal cancer invasiveness.

## Materials and Methods

### Study population

The Institutional Ethics Review Board approval of Tongji hospital was obtained and informed written consent was waived for this retrospective study. All patient information was treated with confidentiality and used only for the purpose of this study. 112 rectal cancer patients were recruited in this study from April 2016 to February 2017. The inclusion criteria were as follows: 1) patients were enrolled in this study consecutively; 2) patients received no previous neoadjuvant treatment with radiotherapy; 3) patients with pathological confirmation of rectal adenocarcinoma after endoscopy-guided biopsy or surgery (the mucinous adenocarcinoma was not included); 4) patients received both fFOV DWI and rFOV DWI imaging. The exclusion criteria were as follows: 1) patients underwent previous chemoradiotherapy treatment before MR or surgery (n = 25); 2) patients could not fulfill MR scanning (n = 13); 3) patients did not receive surgery after MR (n = 15); 4) poor image quality due to obvious motion artifacts or patient movement (n = 10). The final study cohort was comprised of 49 patients (34 males, 15 females; mean age, 56.2 ± 12.2 years; range, 22–82 years).

### MRI examination

All MR examinations were performed by utility of a 3 T scanner (Discovery 750, GE Healthcare, USA) with a 32-channel torso coil. No bowel preparation was given and the intravenous antispasmodic agents were not administered before MR examination. The regular imaging protocol for rectal cancer was as follows: axial T1 FSE (fast spin echo) sequence (TR (repetition time)/TE (echo time), 500/11 msec; section thickness, 5 mm; gap, 1 mm; matrix, 320 × 224; FOV, 380 × 380 mm^2^); axial T2 FSE sequence (TR/TE, 4050/85 msec; section thickness, 5 mm; gap, 1 mm; matrix, 320 × 224; FOV, 380 × 380 mm^2^); and sagittal T2 FSE sequence (TR /TE, 5310/113 msec; section thickness, 3 mm; gap, 0 mm; matrix, 320 × 320; FOV, 250 × 250 mm^2^). The scanning of axial imaging was vertical to the long axis of rectal tumor, identified by sagittal T2 sequence.

Two axial DWI sequences including rFOV DWI and fFOV DWI were performed for all patients. The rFOV DWI scanning parameters were as follows: TR/TE, 4000/75 msec; section thickness, 3 mm; gap, 0 mm; matrix, 128 × 64; FOV, 200 × 100 mm^2^. The fFOV DWI scanning parameters were as follows: TR/TE, 4000/75 msec; section thickness, 3 mm; gap, 0 mm; matrix, 160 × 128; FOV, 400 × 400 mm^2^. Two b values (0 and 800 s/mm^2^) were applied in 3 orthogonal directions. The scan time for both DWI techniques was 2 minutes 32 seconds.

### Quantitative histogram analysis

All the image data referring to rectal cancer were transferred to the PC and analyzed by utility of in-house developed software (Firevoxel, https://files.nyu.edu/hr18/public/projects.html). Two radiologists (with 6 years and 30 years of experience in interpreting abdominal MRI respectively) reviewed all the MR images. The examiners were unaware of histopathological information of patients. Rectal cancers were presented as intraluminal mass or irregular thickening of rectal wall. Rectal lesions were characterized by intermediate high signal on T2WI and high signal on DWI. The examiners performed ROI delineation directly along the border of entire tumor on each slice of DWI images (b = 800 s/mm^2^). T2WI sequence was utilized as reference standard to ensure accurate positioning of ROI. Besides, care was taken to eschew regions of necrotic/cystic components and vessels related to corresponding slice. In addition, the lowest and highest slices of DWI images were excluded for partial volume effects. After all the ROIs were determined on the DWI images, the histogram parameters were automatically calculated by using Firevoxel software and SPSS v. 19.0 (IBM, Armonk, NY, USA). The histogram parameters were comprised of ADCmean (mean ADC value), ADCmax (maximum ADC value), ADCmin (minimum ADC value), 5^th^, 10^th^, 25^th^, 50^th^(or median), 75^th^, 90^th^ and 95^th^ percentiles, skewness, kurtosis, entropy and tumor volume.

### Surgery and pathological evaluation

All the eligible patients underwent curative surgery or palliative operations. The median time interval between surgery and MRI scan was 4 days (range, 1–6 days). The surgical specimens were stained with hematoxylin and eosin for histopathological evaluation. The microscopic histological analysis of resected specimens was performed by a pathologist with 25 years’ experience, who was also blinded to clinical and histopathological information of patients. The histological type and T staging were assessed and recorded according to the 7^th^ edition of TNM system for rectal cancer by the American Joint Committee on Cancer (AJCC).

### Statistical analysis

The interobserver and intraobserver agreements were interpreted utilizing the intra-class correlation coefficient (ICC) test (0.00–0.20, poor; 0.21–0.40, fair; 0.41–0.60, moderate; 0.61–0.80, good; and 0.81–1.00, excellent). The histogram parameters were compared between pT1-2 and pT3-4 stages by independent student t test or the Man-Whitney U-test with Bonferroni correction according to the results of normal distribution test. The Spearman correlation analysis test was performed to evaluate the relationship between histogram parameters and T staging of rectal cancer for both rFOV DWI and fFOV DWI (0.00–0.19, very weak; 0.20–0.39, weak; 0.40–0.59, moderate; 0.60–0.79, strong; 0.80–1.00, very strong). Receiver operating characteristic (ROC) curve analysis was performed to determine the diagnostic perfomance of histogram parameters in distingushing T staging of rectal cancer based on two DWI techniques. We also examined the area under the ROC curve (AUC) to evaluate the diagnostic abilities of ADC values, skewness and kurtosis from rFOV DWI and fFOV DWI. The AUC values of different histogram parameters from both DWI techniques were compared by Z tests.

All the statistical analyses were performed using SPSS v. 19.0 (IBM, Armonk, NY, USA) and MedCalc software (version 12.7.0.0; Mariakerke, Belgium). Values of P < 0.05 were considered significant.

### Compliance with ethical standards

This study involving human participants was approved by the Institutional Review Board (IRB) of Tongji hospital, and we pledged to abide by the declaration of Helsinki (2000 EDITION) in accordance with the relevant medical research rules of China in the study. Written informed consent was waived from all patients. All patient-sensitive information was kept with confidentiality and used only for the purpose of the study.

## Data Availability

The datasets generated and analyzed during the current study are available from the corresponding author on reasonable request.
